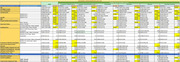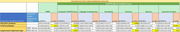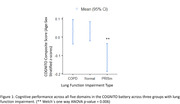# Differential Impact of Pulmonary Impairment Type on Cognitive Function

**DOI:** 10.1002/alz.091275

**Published:** 2025-01-09

**Authors:** Sumedha Mitra, Raghav Prasad, Pravin Sahadevan, Jonas S. Sundarakumar

**Affiliations:** ^1^ Centre for Brain Research, Indian Institute of Science, Bangalore, Karnataka India; ^2^ Centre for Brain Research, Indian Institute of Science, Bangalore (Urban), Karnataka India

## Abstract

**Background:**

Worsening pulmonary health may be independently associated with declining cognitive function. However, the association between specific types of pulmonary impairment with cognitive function is not well understood. The present study aims to determine the differential impact of Chronic Obstructive Pulmonary Disease (COPD) and Preserved Ratio Impaired Spirometry (PRISm) in a rural Indian aging cohort

**Method:**

Data from participants (n = 1223) of the Srinivaspura Aging, Neuro Senescence and COGnition (SANSCOG) cohort were analyzed. Predictive spirometry equations were derived from a similar population. Lung function was classified as normal, COPD or PRISm, based on the Global Initiative for Chronic Obstructive Lung Disease (GOLD) and PRISm criteria, respectively. The Hindi Mental State Examination (HMSE) test was used to assess global cognition, while a comprehensive culturally adapted neuropsychological battery, COGNITO, was used to assess cognitive performance across five domains (visuospatial abilities, language comprehension, attention, memory and executive function). Multivariable regression models, adjusted for confounders (Table 1), were used to determine associations between spirometry parameters and cognitive performance.

**Result:**

We found that PRISm, in comparison to normal lung function, had a significant negative association with the composite COGNITO score (β(95% CI) = ‐0.11 (‐0.18, ‐0.03), p = 0.003) as well as individual domain‐wise scores: attention (β(95%CI) = ‐0.12 (‐0.22,‐0.01), p = 0.02), memory (β(95%CI) = ‐0.12 (‐0.24,‐0.01), p = 0.02) and executive functions (β(95%CI) = ‐0.15 (‐0.28,‐0.02), p = 0.02) (Table 2). No significant associations were noted among between COPD and cognitive test scores.

Forced Vital Capacity (FVC) ratio (FVC/FVC predicted) was significantly associated with HMSE score (β(95%CI) = 0.35 (0.04,0.65), p = 0.02) and the composite COGNITO score (β(95%CI) = 0.14 (0.03,0.24), p = 0.007). However, Forced Expiratory Volume (FEV1) ratio (FEV1/FEV1 predicted) was not significantly associated with HMSE score or the composite COGNITO score.

**Conclusion:**

We observed that PRISm and not COPD was associated with cognitive function, thus suggesting PRISm could be a potential risk factor for dementia (Figure 1). Correlating these findings with brain MRI, could yield findings about their differential impact on brain structure and function.